# *Essential Care for Every Baby:* improving compliance with newborn care practices in rural Nicaragua

**DOI:** 10.1186/s12884-018-2003-y

**Published:** 2018-09-12

**Authors:** Krystle Perez, Jacquelyn Patterson, Jessica Hinshaw, Carlos Escobar, David Parajon, Laura Parajon, Carl Bose

**Affiliations:** 1 Division of Neonatal-Perinatal Medicine, Department of Pediatrics University of Washington, 1959 NE Pacific Street, Box 356320, Seattle, WA 98195 USA; 2 Department of Pediatrics, The University of North Carolina at Chapel Hill, Chapel Hill, NC USA; 3 Amos Health and Hope, Managua, Nicaragua

**Keywords:** Essential Care for Every Baby, Essential newborn care, Implementation, Low and lower middle income countries

## Abstract

**Background:**

Neonatal mortality comprises an increasing proportion of childhood deaths in the developing world. Essential newborn care practices as recommended by the WHO may improve neonatal outcomes in resource limited settings. Our objective was to pilot a *Helping Babies Breathe and Essential Care for Every Baby* (HBB and ECEB) implementation package using HBB-ECEB training combined with supportive supervision in rural Nicaragua.

**Methods:**

We employed an HBB-ECEB implementation package in El Ayote and Santo Domingo, two rural municipalities in Nicaragua and used a pre- and post- data collection design for comparison. Following a period of pre-intervention data collection (June–August 2015), care providers were trained in HBB and ECEB using a train-the- trainer model. An external supportive supervisor monitored processes of care and collected data. Data on newborn care processes and short-term outcomes such as hypothermia were collected from facility medical records and analyzed using standard run charts. Home visits were used to determine breastfeeding rates at 7, 30 and 60 days.

**Results:**

There were 480 institutional births during the study period (June 2015–June 2016). Following the HBB-ECEB implementation package, cord care improved (pre-intervention median 66%; post-intervention shift to ≥85%) and early skin-to-skin care improved (pre-intervention median 0%; post-intervention shift to ≥56%, with a high of 92% in June 2016). Rates of administration of ophthalmic ointment and vitamin K were high pre-intervention (median 97%) and remained high. Early initiation of breastfeeding increased with a pre-intervention median of 25% and post-intervention shift to ≥28%, with a peak of 81% in June 2016. Exclusive breastfeeding rates increased short-term but were not significantly different by 60-days of life (9% pre-intervention versus 21% post-intervention).

**Conclusions:**

The implementation of the HBB-ECEB programs combined with supportive supervision improved the quality of care for newborns in terms of cord care, early skin-to-skin care and early initiation of breastfeeding. The rates of administration of ophthalmic ointment and vitamin K were high pre- intervention and remained high afterwards. These findings show that HBB-ECEB programs implemented with supportive supervision can improve quality of care for newborns.

## Background

Neonatal mortality is the main contributor to childhood mortality in the developing world, accounting for approximately 45% of deaths under 5 years-of-age in 2015 [1]. Over 90% of neonatal deaths occur in low or lower-middle income countries (LICs or LMICs) [2, 3]. Globally, reductions in neonatal mortality have lagged behind reductions in deaths under 5 years-of-age over the last decade. For example, in Nicaragua, the Neonatal mortality rate (NMR) has remained between 15 and 19 per 1,000 live births over the last decade [4]. Although this rate compares favorably to the rate in many LICs and LMICs, significant disparities in newborn outcomes exist between rural and urban areas, and addressing these disparities is a national priority [4].

Nearly all neonatal deaths are attributable to three main causes: complications of preterm birth, intrapartum-related conditions, previously referred to as asphyxia, and infection [5]. Evidence supports the effectiveness of more than 20 perinatal and newborn health practices to reduce neonatal mortality [6]. Most of these practices are included in a bundle of care recommended by the World Health Organization (WHO), referred to as Essential Newborn Care (ENC) [7, 8]. Many health authorities have adopted ENC guidelines as their national standard of care; in the case of Nicaragua, their ministry of health (MINSA) established “*Normativa 108.”* Implementation of ENC reduces mortality in resource-limited areas, but remains challenging to scale up, particularly in remote areas [9–11]. Implementation of evidence based newborn care in these areas may improve with the use of educational programs that are simplified, adaptable to local environments, and deliverable using a train-the-trainer model.

In 2010, a private-public consortium led by the American Academy of Pediatrics (AAP) introduced a simplified, low-cost curriculum for teaching newborn resuscitation in resource-limited areas, *Helping Babies Breathe* (HBB, available at: https://www.aap.org/en-us/advocacy-and-policy/aap-health-initiatives/helping-babies-survive/Pages/Helping-Babies-Breathe.aspx). This program uses small group demonstration, paired learning, and realistic simulations with a mannequin as a teaching model and a train-the-trainer strategy for dissemination that can be easily implemented and replicated in low-resource settings. HBB has demonstrated effectiveness in reducing neonatal mortality and stillbirths in a variety of low-resource settings [12–14]. In 2014, the AAP released *Essential Care for Every Baby* (ECEB, available at: https://www.aap.org/en-us/advocacy-and-policy/aap-health-initiatives/helping-babies-survive/Pages/Essential-Care-Every-Baby.aspx), another simplified, low-fidelity education program that addresses other elements of ENC such as skin-to-skin care, early initiation of breastfeeding (EIBF), administration of ophthalmic ointment and vitamin K. ECEB had not been formally field-tested beyond its initial educational evaluation at the initiation of this project [15].

The objective of this project was to test the effectiveness of education in HBB and ECEB followed by on-site supportive supervision as a strategy to improve compliance with evidence based care practices that have been shown to reduce mortality. Our primary endpoint was exclusive breastfeeding at 60 days, with the hypothesis that the intervention would increase exclusive breastfeeding rates by 30%.

## Methods

### Study design

This interventional, prospective cohort study was conducted in two rural health centers in Nicaragua from June 2015 through June 2016. The study was conducted through a collaboration between the University of North Carolina (UNC), MINSA and AMOS Health and Hope (amoshealth.org), a Nicaraguan non-profit organization with a mission to improve health in rural Nicaragua. The study tested the effectiveness of education in HBB and ECEB followed by on-site supportive supervision by comparing newborn processes of care and outcomes pre-and post-intervention. The need for IRB approval was formally waived by the Office of Human Research Ethics at the University of North Carolina in Chapel Hill, who determined the study did not constitute human subjects research. The Universidad Nacional Autónoma de Nicaragua (UNAN) Facultad de Ciencias Medicas Comite de Etica para Investigaciones Biomedicas (CEIB) in Leon, Nicaragua also reviewed and approved the study. MINSA approved the intervention and allocated employee time for the training.

### Selection of health centers

The two rural health centers for this study were selected as they were among MINSA’s priority centers due to their high NMR, and were also within the AMOS catchment area. One health center was located in the municipality of Santo Domingo in the department of Chontales, and the other in El Ayote of the *Región Autónoma de la Costa Caribe Sur* (RACCS). Health care for each municipality in the region is coordinated by a departmental health system called the *Sistema Local de Atención Integral En Salud* (SILAIS). While El Ayote is in the RACCS department, its health facility is coordinated by Chontales because of its geographic proximity to Chontales.

### Data collection

We collected data on selected newborn care processes and outcomes for all births occurring in our two health centers. Facility data collected included the following processes of care: continuous skin-to-skin care for the first hour after birth, EIBF, ophthalmic ointment administration, cord care and vitamin K treatment. We categorized newborns as having received continuous skin-to-skin care if they were placed skin-to-skin immediately following delivery and remained skin-to-skin for at least 1 h after birth. EIBF was defined as any attempt to breast feed in the first hour, regardless of latch, suckling or transfer of breastmilk. Outcomes included hypothermia, defined by temperature less than 36.5 °C within 1 h following birth, and death prior to discharge from the birth facility. Data were abstracted from each newborn’s medical record by a member of the study staff who was trained in data collection for this study.

We also collected data on exclusive breastfeeding for a small, convenience sample of infants during consecutive home visits at 7, 30 and 60 days after birth. This data was collected by a cadre of pre-existing AMOS community health workers (CHWs) who received additional training in data collection for this study and were supervised by an AMOS staff nurse. Exclusive breastfeeding was defined as the intake of solely human milk, with the exception of vitamins or medicine intake.

The period of active pre-intervention data collection was from June to August 2015; data collection during the intervention occurred from September 2015 through June 2016. The data abstractor entered all data into a digital data collection system created using FileMaker Pro Advanced (version 12, Filemaker, Inc., Santa Clara CA). Stored data was downloaded in batches to a data coordinating center for analysis.

### Intervention

#### Background on the ECEB curriculum

The ECEB curriculum is based on recommendations from the WHO regarding ENC [7, 8]. It emphasizes care following initial resuscitation of the newborn, including skin-to-skin care and EIBF, disease prevention with appropriate eye care, cord care and vitamin K administration, and assessment and classification of the newborn.

For this study, we adapted the ECEB educational material so that it matched national recommendations for care. For example, providers were taught to administer chlorhexidine for cord care and tetracycline for eye care as recommended in MINSA’s policy guidelines on newborn care, called *Normativa 108*. The illustrative human figures in the teaching material were also altered (e.g. skin color and dress) to more closely resemble people in the region.

#### Training Cascade for newborn providers

In July 2015, a three-day HBB/ECEB regional workshop was conducted in Spanish by master trainers. Two representatives attended from each health center in the SILAIS, including two representatives from each of our study health centers. Master trainers included neonatologists from UNC and medical staff from AMOS, with a trainer to trainee ratio of 1:6. Following the regional workshop, providers returned to their health centers with the expectation that they would train their colleagues.

At the two study health centers, the providers who attended the regional workshop trained all newborn care providers in their respective health centers with the help of a supportive supervisor from the AMOS staff. This training took place between August and October 2015, and occurred in smaller installments rather than three consecutive days. All newborn care providers in both study health centers were trained. In total, 7 physicians and 12 nurses were trained in the El Ayote health center, and 5 physicians and 10 nurses were trained in the Santo Domingo health center.

An additional training in HBB and ECEB was conducted in May 2016 after significant staff turnover in April. This training also occurred in smaller installments, similar to the prior facility-based training. Staff turnover occurs in high-need sites throughout the country due to Nicaragua’s two-year mandatory social service year (*servicio social*) for graduates of medical and nursing school, which is the main mechanism by which MINSA staffs remote sites and improves access to healthcare.

#### Training for CHWs

During October and November of 2015, CHWs in five remote rural communities were trained in select portions of ECEB that pertained to education of families such as exclusive breastfeeding, common breastfeeding problems and solutions, and recognition and immediate referral for signs of newborn sepsis (also known as danger signs). The CHWs used an adapted parent guide from the ECEB curriculum to aid in counseling during home visits.

#### Supportive supervision

Supportive supervision was provided by an AMOS public health nurse coordinator (one coordinator split between the two health centers and the CHWs) as well as one AMOS supervision staff member (called *tecnicos)* at each of the two health centers*.* To fulfill her responsibilities, the nurse coordinator made weekly trips to each of the health centers and bi-monthly trips to the rural areas. At the level of the health center, the nurse coordinator’s responsibilities included the following: (1) coordinating with local MINSA leadership to assure support for the program, (2) monitoring performance and data collection by the tecnicos and consolidating data to send to the data collection center, (3) coordinating and leading HBB and ECEB trainings, (4) providing and reviewing run chart data at each health center, and (5) observing approximately 10% of births for adherence to ECEB protocols. In the rural areas, the nurse coordinator trained and supervised the CHWs and reviewed rural health data collected by CHWs.

The tecnicos were former MINSA nursing students who were selected due to their involvement with MINSA, and thus regarded as an integral part of the health team at both health facilities. In addition to data abstraction, they supported the implementation of early newborn care practices by assuring consistent use of the ECEB parent guide for counseling mothers and assisting with continuing training in HBB and ECEB.

#### Additional elements

CHWs conducted home visits at approximately 7, 30 and 60 days after birth in select communities in the Santo Domingo and El Ayote area. CHWs are well-established in the community of El Ayote and are AMOS volunteers who are selected by their own communities. The home visits were a convenience sample conducted by the CHWs, and served the dual purpose of collecting data and continuing the education of families on recommended newborn care. Counseling emphasized the importance of exclusive breastfeeding and strategies to overcome common breastfeeding problems, including mastitis, using the adapted ECEB parent guide. Counseling also reviewed danger signs for the infants that should prompt the family to seek medical care, including jaundice, poor feeding, seizures and other potential signs of illness.

### Outcomes and analytic techniques

The primary outcome for the test of effectiveness was exclusive breastfeeding at 60 days. Assuming a baseline exclusive breastfeeding rate of 35%, the study had 80% power to detect an absolute change in exclusive breastfeeding of 30% or greater with a sample size of 40 births. Exclusive breastfeeding rates were analyzed in aggregate comparing pre- and post-intervention rates at 7, 30 and 60 days, and proportions were compared using a two-sample t-test.

Secondary outcomes included all processes of care and outcomes at the health facility. All processes and hypothermia were analyzed using run charts with rates calculated monthly and compared to the pre-intervention median. Significant changes in measures were identified by shifts and trends. Shifts were defined by six or more consecutive points above or below the median. Trends were defined as a series of five or more points all directed in the same direction [16]. Pre- and post-intervention comparisons were analyzed using June as the month comparator between 2015 and 2016, and proportions were compared using a two-sample t-test.

## Results

During the study period, 480 births occurred in the health centers, representing 55% of the total births in the El Ayote and Santo Domingo regions. Fifty-one of these births occurred prior to the regional workshop, and 77% occurred in the El Ayote Health Center. Ten infants (2% of the cohort) were low birth weight (< 2500 g).

### Processes of care

There was a higher percentage of missing data pre-intervention compared to post-intervention, specifically for skin-to-skin care and EIBF. Seventy-six percent of skin-to-skin care data and 11% of EIBF data was classified as “missing” pre-intervention because there was no information about these processes of care in the chart. Missing data during the post-intervention period declined significantly for skin-to-skin care and EIBF to 24% and 2.5%, respectively. Data was missing for 9% versus 3% for cord care pre- and post-intervention. There was less missing data for the other newborn care processes pre- and post-intervention, 1% versus < 1% for vitamin K administration, 2% versus < 1% for ophthalmic ointment application, and 3.5% versus < 1% for temperature. Missing data was excluded from our analyses.

**Fig. 1:**
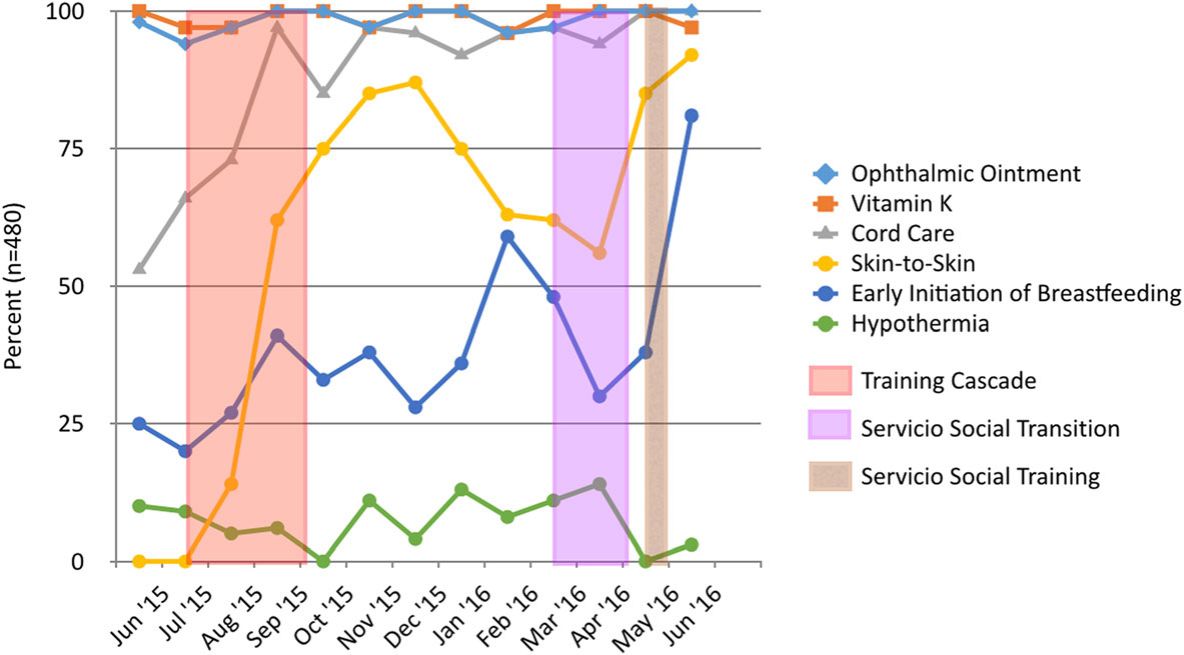
Run Chart Data on Newborn Processes of Care & Outcomes at the El Ayote and Santo Domingo Health Centers

**Fig. 2:**
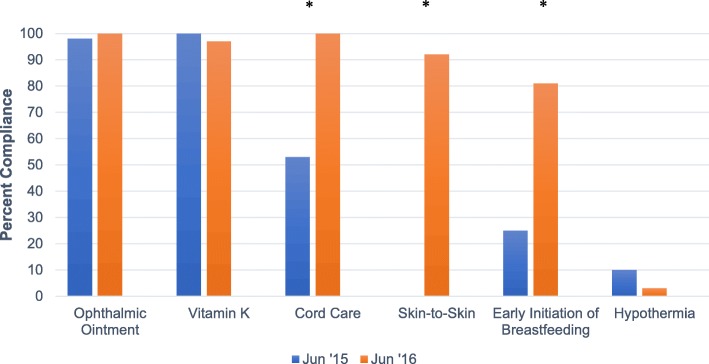
Comparison of Pre- and Post- Training Compliance with Newborn Processes of Care at the El Ayote and Santo Domingo Health Centers

During the period of pre-intervention data collection, ophthalmic ointment and vitamin K administration were provided to nearly all newborns (Fig. 1). Compliance with these processes of care remained high during the post-intervention period. By contrast, cord care increased from a pre-intervention median rate of 66% to greater than 85% after the intervention. Early skin-to-skin care for at least 1 h was not provided prior to the pre-intervention period (median of 0%) with a shift to greater than 56% after the intervention and reached 92% in June 2016. A shift from a pre-intervention median of 25% to a range of 28% to 81% post-intervention was observed for EIBF (Fig. 1). Similar findings were seen when comparing pre- and post-intervention rates of compliance with newborn care practices with significant increase in cord care (from 53 to 100%, *p* < 0.0001), skin-to-skin care (from 0 to 92%, *p* < 0.0001) and early initiation of breastfeeding (from 25 to 81%, *p* < 0.0001) between June 2015 and June 2016 (Fig. 2). Of note, there were significant declines in the proportion of infants with skin-to-skin care for at least 1 h and EIBF from the period from February to April 2016, coinciding with the annual turnover of the servicio social health professional graduates. After additional training for new staff in May of 2016 following the transition in servicio social graduates, skin-to-skin care and EIBF rose to 92% and 81%, respectively, by June 2016.

### Outcomes

No change was observed in the proportion of infants with hypothermia during the pre-intervention period (median 9%) compared to the post-intervention period (range from 0 to 14%), with minimal missing data as noted above. No neonatal deaths occurred prior to discharge from the health centers.

**Fig. 3:**
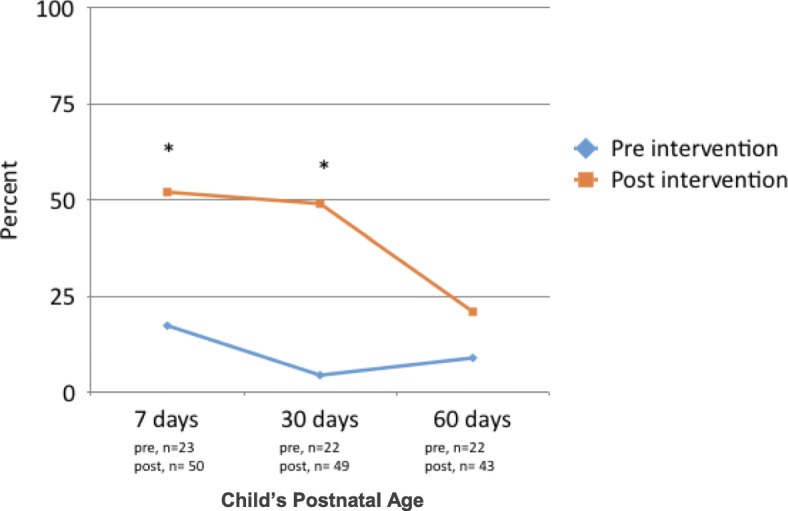
Rates of Exclusive Breastfeeding Pre- and Post-Training

Data describing rates of exclusive breastfeeding were collected during consecutive home visits to 23 mothers who delivered during the pre-intervention period and to 50 mothers who delivered post-intervention. The percentage of mothers reporting exclusive breastfeeding increased from 17 to 52% at 7 days (*p* = 0.007), from 4.5 to 49% at 30 days (*p* = 0.0005) and from 9.1 to 21% at 60 days (*p* = 0.2223) following birth during the post-intervention period compared to the pre-intervention period (Fig. 3). Despite these improvements, exclusive breastfeeding rates steadily declined from 7-days to 60-days after birth for newborns both pre- and post-intervention.

## Discussion

This study tested the impact of training in HBB and ECEB with supportive supervision in two health centers in a rural region of Nicaragua on processes of care and selected outcomes. This intervention resulted in improved rates of compliance with some recommended newborn care practices and stability in rates of compliance with care practices already being consistently performed. Most notable were significant improvements in skin-to-skin care and EIBF. Cord care with chlorhexidine was relatively high pre-intervention and improved post-intervention. Importantly, we observed high pre-intervention compliance with the administration of ophthalmic ointment and administration of vitamin K, and the intervention did not reduce compliance with these practices.

Improvements in select newborn care practices were observed soon after training, and were sustained above the pre-intervention medians throughout the study period. Notably, EIBF and skin-to-skin care declined during the annual turnover of staff with new servicio social graduates replacing existing health professionals with 2 years of clinical experience in the health facilities. Training of new staff in HBB and ECEB improved compliance with both of these practices. This annual turnover of servicio social health professionals highlights the importance of a continuing education program for HBB and ECEB as well as pre-service education.

One newborn care practice of particular interest was EIBF and the subsequent rates of exclusive breastfeeding at 60 days of life. A recent systematic review reported a more than 40% reduction in all cause neonatal mortality and infection-related neonatal mortality when breastfeeding was initiated within 1 h after birth [17]. Exclusive breastfeeding beyond the neonatal period may be protective against infection, specifically diarrheal and respiratory tract infection, and suboptimal breastfeeding contributes to early child mortality in LMICs [18]. The ECEB curriculum has specific content to promote exclusive breastfeeding. Promotion of exclusive breastfeeding may be particularly important in Nicaragua, where rates of exclusive breastfeeding during early infancy are low. Fewer than half of two-month-old infants are exclusively breastfed, and the proportion of exclusively breast fed infants falls with increasing age [4]. Skin-to-skin care and EIBF are associated with an increase in the likelihood of exclusive breastfeeding in infancy [19]. Although rates of skin-to-skin care and EIBF increased after the intervention, rates of exclusive breastfeeding remained undesirably low. Further, although there was a significant increase in the rate of exclusive breastfeeding at 7 and 30 days after birth following the intervention, this difference disappeared by 60 days after birth. There was a steady decline in exclusive breastfeeding rates from nearly 50% at 7 days to 21% at 60 days following birth in the period after the intervention.

We had hoped that early skin-to-skin care, EIBF, and an engagement of CHWs to continue to support breastfeeding would result in improved rates of exclusive breastfeeding beyond the neonatal period. Although our follow-up data suggest that the intervention may improve exclusive breastfeeding, the overall rate of exclusive breastfeeding remains unacceptably low, and any possible improvements appeared to disappear at 60-days. While it is possible that a larger cohort may have demonstrated more significant, sustained improvements, it seems more likely that the beneficial effects of early newborn care practices are outweighed by other, later factors that are barriers to breastfeeding including cultural practices and beliefs. More frequents visits for educational purposes may offset these factors. Quality improvement methodology may be an ideal approach to discover these barriers and potential solutions.

National Nicaraguan data would have predicted at least 6 deaths in our birth cohort, yet no neonatal deaths were reported. Much of our data on neonatal death was collected from facility medical charts, with only 10% of the birth cohort having home visit outcomes at 30 days. As such, we could not capture all deaths that may have occurred after discharge home. In addition, mothers who presented in preterm labor were transferred to a higher-level facility, and therefore were not included in our birth cohort. This may have included a significant number of mothers, evidenced by the low rate (2%) of low birth weight infants in the cohort. Furthermore, given the number of births in our catchment area during the study period, random fluctuation in neonatal mortality could explain our results.

We suspect that a key to the sustained improvements noted was supportive supervision. Supportive supervision has been shown to have a positive effect on performance, with improved motivation and confidence of healthcare workers [20, 21]. Knowledge and skills acquired during HBB and ECEB training may decline over time, as has been observed following other training programs [22, 23]. Based on these observations, we speculate that the clinical coaching as well as monitoring and evaluation provided by the supportive supervisor had a significant impact on processes of care following the training.

An additional factor contributing to the sustained improvements during our study was the support of MINSA. Local adaptation of the HBB and ECEB materials to support the Nicaraguan national standards for newborn care was instrumental in gaining the trust and the commitment of SILAIS leadership at MINSA. MINSA authorized the allocation of time for all health care providers to be trained in HBB and ECEB. In addition, key maternal and child health program staff at the SILAIS level worked with their municipal counterparts at the health facilities to encourage local providers to improve their care practices, as well as ensure that the ECEB posters and appropriate supplies were available in each delivery room. The intersectoral partnership developed with MINSA highlights the importance of collaborative efforts with local health authorities and governments to improve quality of neonatal care and outcomes.

This study had several limitations, most notably the small sample size of births captured overall and in particular for the primary outcome of interest. Additionally, the data from home visits was collected by convenience sample from communities where AMOS was already present. Facility data were collected by abstraction from the medical record, and thus are as reliable as the medical charting. One notable problem was the high rate of missing data for selected practices, particularly during the pre-intervention period. Facility-based data limited our ability to capture neonatal deaths occurring after discharge. Finally, our ability to capture the primary outcome of exclusive breastfeeding was limited by geographic and financial constraints which resulted in only a subset of follow-up home visits. These limitations significantly affect the generalizability of this data to other populations.

Of note is the significant percentage of data missing, particularly skin-to-skin care and EIBF during the pre-intervention period. We cannot definitively conclude that these missing data points reflect that these care practices were not being performed. However, we suspect that skin-to-skin care was, in fact, infrequently being performed prior to the intervention based upon observations by the nurse coordinator, as well as health care worker reports of non-compliance with this practice during the HBB and ECEB Master training. The percentage of missing data did decline post- intervention, demonstrating improvement in documentation following training. However, due to the differences in missing data, pre- and post-intervention data comparison is admittedly made more challenging. Nonetheless, the consistent trends and shifts seen in compliance with some practices such as EIBF and skin-to-skin care seem unlikely to be explained solely by missing data.

Despite these limitations, this study was able to demonstrate aspects of a successful implementation package using HBB and ECEB along with supportive supervision. Implementation of this package led to sustained improvements in ENC practices as recommended by WHO, and resulted in a continued plan for ongoing monitoring and evaluation by in-country collaborators. In addition, the time and effort taken by the leadership of the partners (MINSA, AMOS, LDSC, and UNC) to collaboratively identify shared goals and priorities led to the success of the partnership with MINSA and local health authorities has led to an expansion of the HBB and ECEB supportive supervision program package to the entire department of Chontales.

We believe that our intervention may be a model for improving facility-based newborn care practices. While country-specific policies, demographics and geography may influence successful implementation of similar projects elsewhere, the structure of the current project lends itself well to further implementation in other rural areas. The project highlighted the potential success of private and public partnerships, and a manner in which in-country and international organizations can collaborate successfully. The support of MINSA and their motivation of local providers to improve newborn care practices undoubtedly aided in the improvement in compliance with recommended newborn care practices.

## Conclusions

Our study demonstrates that ENC practices can be improved in facilities in resource-poor communities using HBB and ECEB with in-country supportive supervision. This strategy led to improvements for a number of care practices with low compliance rates prior to training. Although exclusive breastfeeding rates improved in the short-term, improvement in care practices known to increase the likelihood of exclusive breastfeeding during infancy did not result in significantly higher rates at 60-days following birth. Strategies to understand and overcome barriers to exclusive breastfeeding at the community level will be required to improve this outcome.

We describe an implementation strategy that may be a model for programs in other rural, resource-limited areas. We believe three key elements of our strategy were (1) supportive supervision and the promotion of continued monitoring and evaluation within the facilities, (2) a focus on developing a successful partnership with MINSA, and (3) collaborative leadership of each of the partners focused on the common goal of improved neonatal care. While the additional resource for supportive supervision within facilities was initially costly, we believe it was crucial for successful implementation and monitoring and evaluation of HBB and ECEB. Demonstrating the effectiveness of this model in a small region may be a catalyst for a replicable, sustainable model whereby MINSA provides staffing for supportive supervision and training in HBB and ECEB education programs as a strategy for improving quality of care and improving neonatal outcomes.

## Abbreviations

AAP: American Academy of Pediatrics
CHW: Community Health Worker
ECEB: Essential Care for Every Baby
EIBF: Early Initiation of Breastfeeding
ENC: Essential Newborn Care
HBB: Helping Babies Breathe
LIC: Low income countries
LMIC: Lower middle income countries
MINSA: Ministry of Health
NMR: Neonatal mortality rate
SILAIS: *Sistema Local de Atención Integral En Salud*, an entity of MINSA present in each department of Nicaragua
WHO: World Health Organization
